# Identification of a Prognostic Microenvironment-Related Gene Signature in Glioblastoma Patients Treated with Carmustine Wafers

**DOI:** 10.3390/cancers14143413

**Published:** 2022-07-14

**Authors:** Ivana Manini, Emiliano Dalla, Vera Vendramin, Daniela Cesselli, Carla Di Loreto, Miran Skrap, Tamara Ius

**Affiliations:** 1 Institute of Pathology, University Hospital of Udine, 33100 Udine, Italy; daniela.cesselli@uniud.it (D.C.); carla.diloreto@uniud.it (C.D.L.); 2 Department of Medicine, University of Udine, 33100 Udine, Italy; emiliano.dalla@uniud.it; 3 IGA Technology Services, SRL, 33100 Udine, Italy; vvendramin@igatechnology.com; 4 Neurosurgery Unit, Department of Neurosciences, University Hospital of Udine, 33100 Udine, Italy; skrap.asufc@sanita.fvg.it (M.S.); tamara.ius@asufc.sanita.fvg.it (T.I.)

**Keywords:** glioblastoma, carmustine wafers, tumor microenvironment, patient’ derived in vitro model, transcriptomics

## Abstract

**Simple Summary:**

Carmustine wafer (CW) implantation into the resection cavity of patients operated for glioblastoma (GBM) was approved as an adjuvant treatment before the Stupp Protocol. Although contrasting clinical results limited its use, our retrospective study on 116 GBM treated with CW showed a significant benefit in terms of OS in a subgroup of patients. Since GBM growth, progression, and drug resistance are supported by the surrounding environment, and since the tumor microenvironment (TME) is the source of druggable targets, we hypothesized that the TME of patients who benefited from CW could have different characteristics compared to patients who did not show any advantage. Exploiting a human in vitro model of glioma microenvironment and a transcriptomic approach, we found a different gene signature suggesting the importance of developing in vitro models that mimic the properties of human cancers and that can help to study individual patient characteristics at the cellular and molecular level.

**Abstract:**

Despite the state-of-the-art treatment, patients diagnosed with glioblastoma (GBM) have a median overall survival (OS) of 14 months. The insertion of carmustine wafers (CWs) into the resection cavity as adjuvant treatment represents a promising option, although its use has been limited due to contrasting clinical results. Our retrospective evaluation of CW efficacy showed a significant improvement in terms of OS in a subgroup of patients. Given the crucial role of the tumor microenvironment (TME) in GBM progression and response to therapy, we hypothesized that the TME of patients who benefited from CW could have different properties compared to that of patients who did not show any advantage. Using an in vitro model of the glioma microenvironment, represented by glioma-associated-stem cells (GASC), we performed a transcriptomic analysis of GASC isolated from tumors of patients responsive and not responsive to CW to identify differentially expressed genes. We found different transcriptomic profiles, and we identified four genes, specifically down-regulated in GASC isolated from long-term survivors, correlated with clinical data deposited in the TCGA–GBM dataset. Our results highlight that studying the in vitro properties of patient-specific glioma microenvironments can help to identify molecular determinants potentially prognostic for patients treated with CW.

## 1. Introduction

The current standard therapy for glioblastoma (GBM), represented by maximal surgical resection combined with chemo- and radiotherapy, offers only a palliative treatment since the median overall survival (OS) is less than 2 years [1], and the 5-years OS after diagnosis is only around 7% [2,3]. It is now well accepted that GBM growth, progression, and resistance to therapy are not only supported by glioma initiating cells (GICs) and tumor cells but also by their interaction with the surrounding environment, defined as tumor microenvironment (TME) [4,5,6]. TME contains various types of non-tumoral cells such as endothelial cells, stromal cells, pericytes, immune cells, and components of the extracellular matrix (ECM), cooperating with tumor cells to create a microenvironment able to promote proliferation, invasion, angiogenesis, and the immune suppression of GBM. Carmustine (1,3-bis(2-chloroethyl)-1-nitrosourea) wafer implants were approved by the FDA for new High-Grade Gliomas (HGGs) in 2003 as adjuvant therapy, preceding the Stupp protocol [7,8]. These biodegradable wafers are applied directly to the tumor resection cavity intraoperatively, providing a slow release of the drug over two weeks, overcoming the Blood–Brain Barrier (BBB), and enabling a better in situ treatment and decreased toxicity [9].

How CW affects the TME, however, is poorly investigated. A recent work [10] described a significant increase in CD8+ and CD68+ cells in the brain of patients who received wafers insertion in comparison to patients without CW. The infiltration of CD8+ cytotoxic T cells after CW implantation is suggestive of stimulation of the local immune response, counteracting the tumors, and it is found to be associated with a better prognosis in gliomas [11]. By contrast, a study performed on healthy monkey brains reported the absence of inflammatory cells around the site of CW insertion, indicating that the immune response engaged by CW is activated only in the presence of the tumor [12].

Despite this evidence, the widespread use of CWs has been limited due to the lack of satisfactory phase III studies [13,14,15] and the presence of contrasting clinical results; some show efficacy in prolonging patients’ survival, while others discourage its use because of toxicity [16,17,18]. Finally, CWs are quite expensive, and their use precludes the enrolment in subsequent trials. In the neurosurgery unit of the Academic Hospital of Udine (ASUFC), a retrospective mono-institutional study on a cohort of 116 patients with HGG treated with CW at surgery showed that a subgroup of 20 (17%) exhibited a long-term survival (OS ≥ 36 months) with a low rate of side effects [19]. Compared to the other patients (OS ≤ 36 months), these long-surviving patients were characterized by a significantly reduced age, an increased proportion of cases that were found to exhibit a methylated MGMT, and successful total tumor resection. This investigation supports a potential survival benefit, conferred via CW treatment, for selected good responder patients and highlights the need to tailor carmustine wafer implantation in groups of patients that will really benefit from it after evaluating individual differences at the clinical and molecular levels. In the last years, many efforts have been made to improve GBM management and to tailor therapies to the characteristics of each patient [20], the most used approaches are represented by genomic analyses of the tumor, which help histology in refining glioma diagnosis and prognosis [21,22,23], and by the development of in vitro models that mimic the properties of human cancers and that are instrumental in deeply understanding tumor biology and in identifying new targets for treatments, both on cancer cells and their surrounding TME [24].

Since the TME is a driver in the development of human tumors, by modulating the acquisition of hallmarks of cancer (e.g., tumor-promoting inflammation, immune evasion, and invasion) [25], here we employed an in vitro model of the tumor microenvironment to identify potential targets predictive of the response to CW implantation at the cellular level. We previously established a protocol to isolate a population of human adult stem cells, or glioma-associated stem cells (GASC), from human low- and high-grade gliomas [26]. GASCs are characterized by an undifferentiated mesenchymal phenotype, clonogenicity, and multipotency in vitro. This cell population is devoid of tumor-initiating properties in vivo (data not published), and it does not show genetic aberrations characterizing the tumor of origin. Nevertheless, GASCs are characterized by the ability to grow in an anchorage-independent way and to support the biological aggressiveness of tumor cells, including their motility, in vitro [27]. We have also already demonstrated that GASCs interact with tumor cells (both Glioma Stem Cells (GSC) and immortalized tumor cell lines) through the release of exosomes [27,28]. For these reasons, GASCs represent bona fide Glioma Stromal Cells residing in the tumor microenvironment. Furthermore, we have reported that the transcriptomic profiling of GASC allows for the identification of prognostic gene signatures in low-grade and high-grade gliomas, suggesting their clinical relevance [29,30,31].

In the present work, we employed a transcriptomic approach to describe the gene expression profile of GASC isolated from tumors explanted before CW implantation. We chose to analyze and compare GASC derived from long-term (GASC-LS, OS ≥ 30 months and mean PFS of 24 ± 9 months) and short-term (GASC-SS, OS ≤ 14 months and mean PFS of 8 ± 2 months) survivors. We found 374 differentially expressed transcripts, and by focusing the analysis on a panel represented by 78 protein-coding genes, we observed that GASC-LS were characterized by a statistically significant down-regulation of four clinically relevant genes. These findings suggest that the TME could provide important biomarkers allowing to identify the group of patients for which a CW-based therapy is recommended over traditional approaches.

## 2. Materials and Methods

### 2.1. Surgical Procedure

Tumor tissues were collected from patients (age ≥ 18 years) affected by newly diagnosed GBMs arising de novo. All of the patients underwent surgery at the Neurosurgical Department of the Academic Hospital Udine (ASUFC). The criteria for CW implantation have been previously described [19]. Briefly, all of the patients did not undergo any previous surgery nor any adjuvant therapy and exhibited a closed surgical cavity. Multifocal lesions and/or lesions extending across the corpus callosum were excluded. The patients had at least 12 months of follow-up and were followed with neurological assessment and post-operative MRI every 4 months.

CWs were implanted in all patients after surgical tumor removal and the intraoperative pathological confirmation of GBM. They were not implanted when tumor removal required the creation of a large opening of a ventricle and/or the basal cistern. The research was approved by the local ethics committee (Parere 196/2014/Em). Written informed consent was obtained from all of the patients. Clinical investigations were conducted according to the principles expressed in the Declaration of Helsinki.

### 2.2. Isolation and Culture of Glioma Associated Stem Cells (GASC)

Glioma-associated stem cells (GASC) were isolated from surgical samples of a removed tumor before CW implantation and maintained in vitro, applying, with minor modifications, a protocol optimized for culturing multipotent adult stem cells from normal and neoplastic human tissues [26]. Briefly, the GBM fragments were first mechanically disaggregated with scalpels and then enzymatically dissociated in a 0.025% Collagenase type II solution (Worthington, Columbus, OH, USA) in Joklik-modified Eagle’s Medium (Sigma-Aldrich, Saint Louis, MO, USA), for 5 min at 37 °C. Collagenase activity was stopped by adding 10% Fetal Bovine Serum in Joklik-modified Eagle’s Medium (Sigma-Aldrich, Saint Louis, MO, USA). The cell suspension was centrifuged at 500× *g* for 10 min after less than 40 μm in diameter. Then, 2.0 × 10^6^ freshly-isolated human cells were plated onto 100-mm diameter human fibronectin (Sigma-Aldrich, Saint Louis, MO, USA)-coated dishes (BD Falcon, Franklin Lakes, NJ, USA) in an expansion medium that was composed as follows: 60% low glucose DMEM (Invitrogen, Waltham, MA, USA), 40% MCDB-201, 1 mg/mL linoleic acid-BSA, 10^−9^ M dexamethasone, 10^−4^ M ascorbic acid-2 phosphate, 1× insulin-transferrin-sodium selenite (all from Sigma-Aldrich), 2% fetal bovine serum (StemCell Technologies, Cambridge, UK), 10 ng/mL of human PDGF-BB, 10 ng/mL of human EGF (both from Peprotech EC, London, UK). The clinical characteristics of all of the patients from which GASC was analyzed in the study were isolated and are reported in Supplementary Table S1.

### 2.3. GASC RNA Extraction, Library Preparation and Sequencing

We analyzed the GASC cell lines derived from patients who showed an OS ≥ 30 months (GASC-LS; n = 3) and an OS ≤ 14 months (GASC-SS; n = 2) evaluated after CW implantation at the time of surgery. The total RNA was extracted using the RNAeasy mini kit (Qiagen GmbH, Hilden, Germany) according to the manufacturer’s instructions. The Universal Plus mRNA-Seq kit (Tecan Genomics, Redwood City, CA, USA) was used for library preparation following the manufacturer’s instructions (library type: fr-secondstrand). The RNA samples were quantified and quality tested by Agilent 2100 Bioanalyzer RNA assay (Agilent Technologies, Santa Clara, CA, USA) or by Caliper LabChip GX (PerkinElmer, Waltham, MA, USA). The final libraries were checked with both Qubit 2.0 Fluorometer (Invitrogen, Carlsbad, CA, USA) and Agilent Bioanalyzer DNA assay or by the Caliper LabChip GX (PerkinElmer, Waltham, MA, USA). The libraries were then prepared for sequencing and sequenced using the single-end 150 bp mode on a NovaSeq 6000 (Illumina, San Diego, CA, USA). Quality control for the raw sequencing reads was performed using the FastQC (v0.11.9) [32] and MultiQC (v1.09) software [33]. Quality, adapters, and contamination filtering were performed using the Trimmomatic command-line tool [34]. The processed reads were aligned to the NCBI GRCh38 human reference using STAR (v2.7.1a) [35]. Transcripts assembly and the number of reads per gene were determined using Stringtie (v1.3.6) [36]. Differentially expressed genes were identified using the DESeq2 (v1.26.0) R/Bioconductor package [37], applying the Wald test; we adjusted for multiple hypothesis testing by employing the Benjamini–Hochberg correction, considering as statistically significant the results having abs(log2FC) ≥ 1, FDR < 0.05. Extended gene annotations (including HGNC gene symbol, description, and transcript type) were obtained using the biomaRt (v2.42.0) R/Bioconductor package [38].

### 2.4. Functional Enrichment Analysis

Significantly up- and down-regulated genes identified in the differential gene expression analysis were used to query the MSigDB database, investigating statistically relevant biological associations. Specifically, we examined the C4, C6, CP, and GeneOntology: BiologicalProcess, H, and IMMUNESIGDB ontologies: C4 gathers cancer-related signatures originated by the data mining of the large microarray data, C6 collects signatures related to pathways deregulated in cancer, CP includes data from canonical pathways (i.e., KEGG, BIOCARTA, Reactome, PID, and WikiPathways), and IMMUNESIGDB contains gene signatures derived from chemical and genetic perturbations of the immune system. The top-20 significantly-enriched (FDR *q*-value < 0.05) genesets were retrieved.

### 2.5. RT2 Profiler™ PCR Array

Seventy-Eight genes were selected to create a customized RT2 Profiler PCR Array (CLAH43115; Qiagen Gmbh, Germany). cDNA was synthesized using the RT2 First Strand Kit (Qiagen) following the manufacturer’s instructions. According to the manufacturer’s protocol, real-time PCR was performed using the RT2 Profiler PCR Arrays in combination with the RT2 SYBR Green/ROX PCR Master Mix (Qiagen). A 102-μL cDNA synthesis reaction volume was mixed with 2 × RT2 SYBR Green Mastermix and RNase-free water to obtain a total volume of 2700 μL. Subsequently, 25 μL of the PCR component mix was dispensed into each of the 96-well PCR arrays. The cycling program, performed on a Roche LightCycler 480, comprises three steps: 95 °C for 10 min for 1 cycle, 45 cycles at 95 °C for 15 s, and 60 °C for 60 s. Data analyses were performed using the manufacturer’s software (https://geneglobe.qiagen.com/us/analyze/ (accessed on 20 January 2022)), which calculates the fold change/regulation of the investigated genes using the delta Ct (∆Ct) method. Briefly, ∆Ct was calculated between each gene and an average of the housekeeping genes (*ACTB*, *B2M*, *GAPDH*, *HPRT1*, and *RPLP0*). Then, ∆∆Ct was extrapolated as the difference between ∆Ct of genes in the tested group (GASC-LS) and ∆Ct of the same genes in the control group (GASC-SS). Finally, fold change was calculated using 2^(−∆∆Ct)^ formula.

### 2.6. Differential Gene Expression Analysis in the TCGA-GBM Dataset

The significantly differentially expressed genes identified with the RT2 Profiler array were tested by comparing their expression levels in the TCGA-GBM dataset (n = 163) with respect to the TCGA-GTEx matched normal samples (n = 207). The data were obtained from the GEPIA web server (http://gepia.cancer-pku.cn/index.html, last accessed on 1 February 2022) and summarized as boxplots; |Log2FC| Cutoff: 1; *p*-value Cutoff: 0.01.

### 2.7. Survival Analysis

The genes differentially expressed according to the RT2 profiler array underwent a survival analysis to define their prognostic value in the TCGA–GBM dataset on a single gene basis in terms of overall survival (OS), using the GEPIA2 web server (http://http://gepia2.cancer-pku.cn/, last accessed on 1 February 2022). Afterward, we defined a four-genes signature and tested it in the same way. Median or tertile cutpoints were used to stratify patients.

## 3. Results

### 3.1. Transcriptomic Characterization of GASC through RNA-Seq

The gene expression profile of GASC isolated from GBM of patients who underwent CW implantation during surgery before the standard Stupp protocol was evaluated by transcriptomic analysis. We compared three GASC cell lines derived from patients with OS ≥ 30 months, ranging from 30 to 43 months, and median PFS of 33 months, ranging from 30 to 41 months, (GASC-LS) and two GASC cell lines derived from patients with OS ≤ 14 months, ranging from 12 to 16 months, and median PFS of 6 months, ranging from 4 to 8 months (GASC-SS). Supplementary Table S2 displays the 374 differentially expressed transcripts (protein-coding, pseudogenes, and lncRNA), 221 up- and 153 down-regulated in GASC-LS compared to GASC-SS. The significantly up- and down-regulated genes identified in the differential gene expression analysis were used separately to query the MSigDB database, investigating statistically relevant biological associations. Specifically, we examined five major collections of gene sets present in the database: GeneOntology: BiologicalProcess, cancer-related signatures, pathways deregulated in cancer, canonical pathways (i.e., KEGG, BIOCARTA, Reactome, PID, and WikiPathways), and gene signatures deriving from chemical and genetic perturbations of the immune system. The top 20 significantly enriched (FDR q-value < 0.05) genesets for up (Table 1) and down-regulated genes (Table 2) in GASC-LS were reported. The most represented enriched terms found between genes up-regulated in GASC-LS were associated with neurogenesis, cell and neuron differentiation, central nervous system development, and secretion, while the down-regulated genes were associated with cell–cell signaling, defense response, and the regulation of the immune system. Interestingly, both up- and down-regulated genes were associated with two common biological processes, namely regulation of ion transport and regulation of transport.

### 3.2. A Customized RT-PCR Array Identified Four Genes Specifically Modulated in GASC-LS

**Figure 1:**
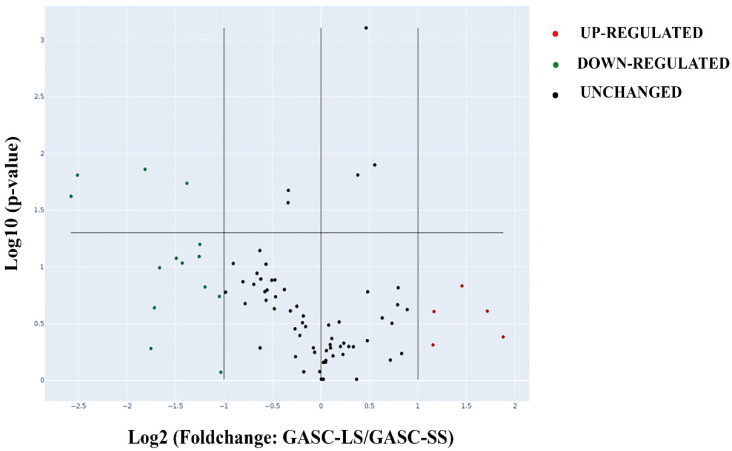
Comparison of the gene expression profile of GASC-LS (n = 6) versus GASC-SS (n = 5), using a customized RT2 PCR Array. Volcano plot shows the gene expression changes of 78 protein-coding transcripts analyzed using the RT2 Profiler™ PCR Array. The volcano plot highlights significant gene expression changes by plotting the log2 of the fold changes in gene expression on the *x*-axis versus their statistical significance on the *y*-axis. The center vertical line indicates unchanged gene expression, while the two outer vertical lines indicate the selected fold regulation threshold (abs (≥2)). The horizontal line indicates the selected *p*-value threshold (*p* ≤ 0,05). Genes with data points in the far upper left (down-regulated, green dots) and far upper right (up-regulated, red dots) sections meet the selected fold regulation and *p*-value thresholds.

To give insight into the differences between GASC-LS and GASC-SS transcriptomic profiles, we manually selected, among the 374 differentially expressed genes obtained by RNA-seq, transcripts with abs(logFC) ≥ 2 described as “protein-coding”, and we identified 78 genes. To evaluate their expression by real-time PCR, we created a customized RT2 profiler PCR array panel, and we validated their expression on the same cell lines used for RNA-seq and extended the analysis to three other GASC-LS (OS ≥ 30 months and mean PFS of 24 ± 9 months) and three other GASC-SS (OS ≤ 14 months and mean PFS of 8 ± 2 months). Figure 1 shows the volcano plot representing the changes in gene expression, derived from the comparison of six GASC-LS with five GASC-SS by plotting the log2 of the fold changes (greater than 2, red dots, or less than 2, green dots) on the *x*-axis versus their statistical significance (*p* ≤ 0.05). For most of the genes in the array, we did not find any statistically significant difference. However, four genes resulted in significantly down-regulated (green dots in the upper left side of the graph) in GASC-LS: ALPL (Alkaline Phosphatase), GPR68 (G protein-coupled receptor 68), NETO1 (neuropilin and tolloid-like 1), and VGF (or VGF nerve growth factor inducible), (Table 3).

### 3.3. Clinical Correlation of Genes Down-Regulated in GASC-LS

**Figure 2:**
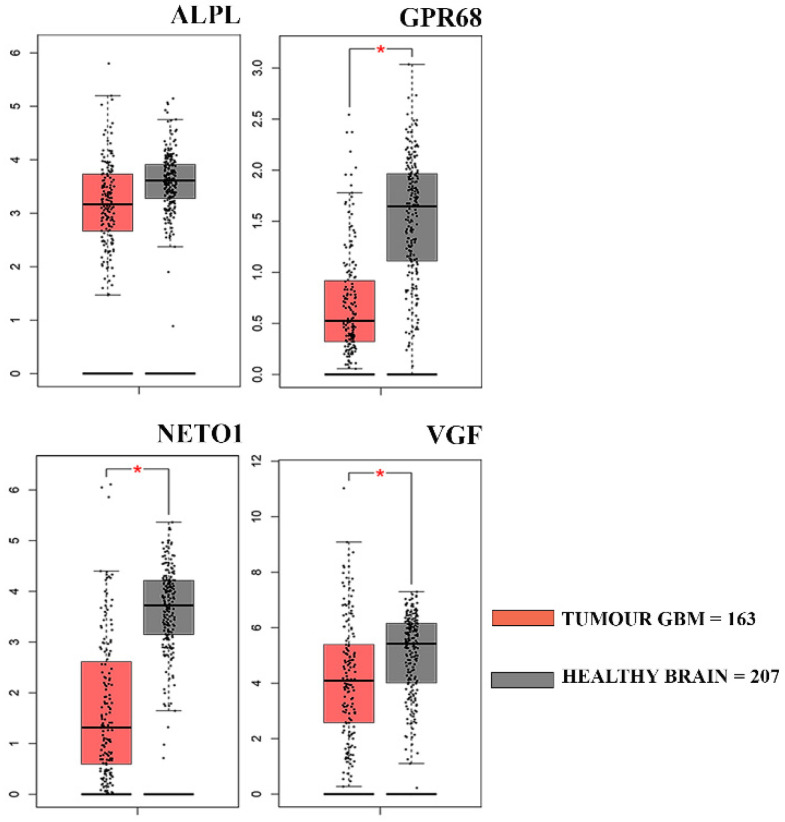
Gene expression profiling of the four genes down-regulated in GASC-LS in the TCGA-GBM RNA-seq dataset. Box plots represent the gene expression levels in the TCGA-GBM dataset (n = 163) and in the TCGA-GTEx matched normal samples (n = 207) of the genes with a significant down-regulation in GASC-LS, identified with the RT2 Profiler array. Data were obtained and plotted from the GEPIA web server (|Log2FC| Cutoff: 1; * *p*-value < 0.01).

**Figure 3:**
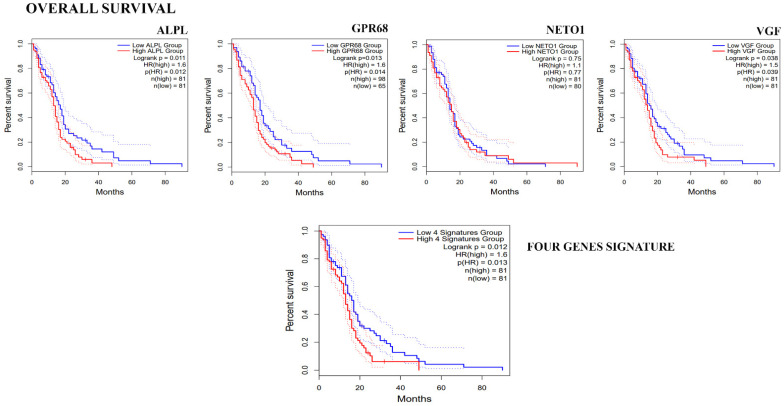
Clinical relevance of the 4 genes down-regulated in GASC-LS. The prognostic value of ALPL, GPR68, VGF, and NETO1 was assessed independently in the TCGA-GBM RNA-seq dataset evaluating the Overall Survival (OS) and was represented by Kaplan–Meier plots. Afterward, the analysis was repeated considering the four-genes signature (median expression). Patients were stratified based on the optimal cut-point.

To investigate if the four genes significantly down-regulated in GASC-LS compared to GASC-SS, according to the RT2 profiler array, could have clinical relevance, we evaluated their expression in 163 GBM patients included in the Cancer Genome Atlas GBM dataset (TCGA-GBM). To this aim, we queried the GEPIA web server comparing the TCGA RNA-Seq data of GBM patients to that of 207 TCGA/GTEx matched normal samples. As shown in Figure 2, ALPL, GPR68, NETO1, and VGF were all up-regulated in normal brain tissue, suggesting their specificity for non-tumoral cells-microenvironmental, as GASCs were described. Afterward, we performed a survival analysis with these genes to establish their prognostic value in the TCGA-GBM dataset on a single gene basis in terms of overall survival (OS, defined as the time between cancer diagnosis and death for any cause), using the GEPIA2 web server. We observed that patients with low expression of ALPL, GPR68, and VGF show a significantly longer OS. A similar, although not statistically significant, the trend was reported for NETO1. Moreover, these four genes, when taken together as a whole signature, retained their prognostic value (Figure 3). When we repeated the same type of analysis in terms of PFS, we found that the low expression of these genes is correlated with a longer PFS, although the correlation is statistically significant only for GPR68 (Supplementary Figure S1). Altogether, these results suggest the identification of four genes that should be specifically expressed in cells of the GBM microenvironment. Interestingly, they were found to be down-regulated in patients that, after CW treatment, have shown an OS ≥30 months, thus suggesting the presence of a less tumor-supporting environment.

## 4. Discussion

Outside the standard of care for GBM treatment, carmustine wafer insertion into the resection cavity has been approved as adjuvant therapy, giving a bridge between surgery and the initiation of the Stupp protocol [7,8]. The benefit of CW implantation is debated in the literature due to reported contrasting results, side effects, its costs, and the exclusion of treated patients from other trials [39,40]. However, a retrospective study performed in the neurosurgery unit of the Academic Hospital of Udine (ASUFC) found a category of patients that really benefited from CW, in terms of OS (≥36 months), with negligible side effects [19]. In the present work, we took advantage of case studies treated with CW and of an in vitro model of the GBM microenvironment, represented by the GASC cell lines, to give more insights into which determinants could influence the response to CW at the cellular level. We initially performed an explorative transcriptomic analysis of GASC-LS and GASC-SS to find out which differences characterized the TME of patients who showed a long-term OS (≥30 months) with respect to those who did not show any improvement of OS (≤14 months), after receiving CW, at the time of surgery. We found that 374 genes were differentially expressed in GASC-LS compared to GASC-SS in a statistically significant manner. The functional enrichment analysis revealed that the most represented enriched terms found in up-regulated genes in GASC-LS were associated with neurogenesis, cell and neuron differentiation, and central nervous system development. These findings possibly indicate that the microenvironment of GBM responsive to CW has an asset more differentiated and more committed to administrating central nervous system basic functions rather than supporting tumor growth. Moreover, the down-regulated genes were associated with cell–cell signaling and the regulation of the immune system, describing a poor communicative TME, thus suggesting a possible interference in the cross-talk with the tumor, which is thought to support its growth and recurrence. Interestingly, both up- and down-regulated genes in GASC-LS were associated with two common pathways: the regulation of ion transport and regulation of transport, represented mainly by genes coding for ion channels. The transport of ions across the cell membrane is a fundamental process for maintaining normal cellular functions and activity (cell cycle, cell death, cell volume regulation, and proliferation) [41,42]. Moreover, it is well accepted that cancers of the nervous system cross-talk within the local tumor microenvironment. Communication between cancer cells and neurones is driven by synapses through the release of neurotransmitters and voltage-gated mechanisms to regulate cancer cell growth [43,44]. Furthermore, glioma cells can electrically integrate into neural circuits through neuro-glioma synapses [43]. Ion channels are also involved in pathways modulating tumor vascularisation and tumor-immune cell interactions [45]. It is likely that the ion channels’ activity and their dysregulations underlie several known hallmarks of glioma, such as proliferative capacity, apoptotic escape, and invasion, and they are now regarded as new therapeutic targets [46,47,48,49]. The finding that most of the genes differentially regulated in GASC-LS and GASC-SS belong to the “ion channels” category suggests that they play an important role in our model, worthy of further investigation. To analyze the expression profile of GASC-LS and GASC-SS in more detail, we selected 78 genes with an abs(logFc) ≥ 2, described as “protein-coding”, and we performed a gene expression assay using a customized RT2-Profiler PCR array. Most of these genes are not differentially expressed between GASC-LS and GASC-SS, except for ALPL, GPR68, NETO1, and VGF, which were significantly down-regulated in GASC-LS. The role of these four genes in the GBM microenvironment is unknown, although their expression has been described in other types of tumors. ALPL is reported as a marker of embryonic stem cells, and it is highly expressed in induced-Pluripotent Stem Cells (iPSCs) obtained from human fibroblasts [50]. Moreover, a role of ALPL was described in malignant leukemia, in which changes in its levels can be used to identify rare populations of highly refractory malignant cells [51].

GPR68 is a GPCRs (G protein-coupled receptor)’s family member. GPCRs modulate signal transduction pathways and cellular processes that are critical for physiological functions [52] and for the initiation and progression of tumors [53,54]. GPR68 is a proton-sensing G-protein-coupled receptor regulated by extracellular acidity and involved in the regulation of a variety of cellular functions. Acidosis is considered a hallmark of the tumor microenvironment. Multiple factors, such as hypoxia, inflammation, and glycolytic cell metabolism, contribute to creating an acidic TME [55,56,57], and it has been shown that acidosis modulates cell proliferation, apoptosis tumor progression, metastasis, anti-tumor immunity, and angiogenesis [58,59]. The expression of GPR68 is highly up-regulated in numerous types of cancer, including prostate, colon and pancreatic tumors, melanoma, myelodysplastic syndrome, and medulloblastoma [60]. Emerging evidence has revealed that GPR68 may play crucial roles in the biology of pancreatic ductal adenocarcinoma (PDAC). Its activation induces Cancer-Associated Fibroblast (CAFs) to acquire an enhanced tumor-promoting phenotype and promotes PDAC cell proliferation [61]. NETO1 is a gene coding for a recently described protein involved in the modulation of glutamate receptor activity [62,63]. In particular, Neto1 is an auxiliary subunit modulating the gating properties of Kainate receptors (KARs), a subfamily of ionotropic glutamate receptors that mediate excitatory transmission and regulate neurotransmitter release at the pre- and post-synaptic level [64,65]. Although Neto1 was not previously described in glioma, KARs have been implicated in epilepsy and other neuropsychiatric conditions [66,67]; moreover, the high expression of Neto1 was associated with metastatic ovarian carcinomas, in which it increases the migration and invasion capability of cancer cells, modulating actin cytoskeletal dynamics [68]. For these reasons, investigating their implication and modulation on highly invasive tumors such as GBM would be of great interest. Finally, VGF encodes a neuropeptide precursor expressed in several types of neurons in the central and peripheral nervous system [69,70,71,72]. The role of VGF in tumors is poorly described. In lung cancer, it seems to be associated with the acquisition of resistance to EGFR inhibitors [73], and in breast cancer, it is involved in the epithelial-to-mesenchymal transition [74]. A recent study identified VGF as a key player in the bidirectional influence between Glioma stem cells (GSC) and their differentiated progeny (DGCs, differentiated glioma cells) [75]. In the model described by Wang et al., VGF contributes to glioma progression by maintaining self-renewal and proliferation of both GSC and DGCs. Further investigation will clarify if the different expression of VGF, in our model, correlates with a different level of communication intervening into the GBM’s TME of patients with long-term and short-term survival, causing an opposed tumor supporting action. Since the present study is only descriptive and performed on a restricted number of cases, we attempted to improve our results by evaluating the expression of ALPL, GPR68, NETO1, and VGF in the Cancer Genome Atlas GBM dataset (TCGA-GBM). We found that these four genes are predominantly expressed in healthy brains rather than in GBM. Moreover, a survival analysis highlighted that the low expression of each single gene was associated with patients showing a significantly longer OS. This result was also confirmed when ALPL, GPR68, NETO1, and VGF were evaluated together. Moreover, the low expression of these four genes showed a correlation with a longer PFS, although in a not statistically significant manner, thus confirming their possible clinical relevance. In summary, here, we described the gene expression profile of an in vitro model of GBM’s TME, represented by the GASC cell lines. We found that GASC-LS displayed a profile not directly correlated with response to therapy but suggestive of a less tumor-promoting action. Of course, future studies will be required to better explain these results and to clarify the mechanistic functions of ALPL, GPR68, NETO1, and VGF in the GBM TME and how they can mediate the success of CW application in the tumor resection cavity.

## 5. Conclusions

The efficacy of carmustine wafers in the treatment of GBM is a still debated question, and the identification of patients who may really benefit from them is needed. Here, we showed that studying an in vitro model of patient-derived TME using a transcriptomic approach highlighted differences in terms of gene expression between patients selected for the response to CW. Deepening the knowledge of these differences will help to explain how the TME molecular landscape is connected with the success of CW application.

## Figures and Tables

**Table 1:** The Top 20 significantly enriched MSigDB pathways associated with the specific lists of genes up-regulated in GASC-LS. The table reports the overall number (#) of genes involved in the annotated pathways, the number (#) of genes provided as input, and the associated *p*-value.

GASC-LS UP			
Gene Set Name	#Genes in Gene Set	#Genes in the Overlap	*p*-Value
REGULATION OF ION TRANSPORT	1314	27	4.36 × 10^−10^
NEUROGENESIS	1613	30	4.52 × 10^−10^
NOTCH_SIGNALING_PATHWAY	186	11	2.59 × 10^−9^
REGULATION_OF_TRANSPORT	1730	29	9.28 × 10^−9^
REGULATION_OF_CELL_DIFFERENTIATION	1618	28	8.74 × 10^−9^
NEURON_DIFFERENTIATION	1357	25	1.77 × 10^−8^
BMI1_DN_MEL18_DN.V1_DN	147	9	5.55 × 10^−8^
SECRETION	1464	25	7.61 × 10^−8^
MORF_DCC	112	8	9.39 × 10^−8^
BEHAVIOR	541	15	9.61 × 10^−8^
POSITIVE_REGULATION_OF_ION_TRANSPORT	659	16	2.14 × 10^−7^
MORF_EPHA7	139	8	4.96 × 10^−7^
POSITIVE_REGULATION_OF_TRANSPORT	882	18	4.66 × 10^−7^
POSITIVE_REGULATION_OF_MULTICELLULAR_ ORGANISMAL_PROCESS	1397	23	4.96 × 10^−7^
POSITIVE_REGULATION_OF_DEVELOPMENTAL_ PROCESS	1284	22	4.62 × 10^−7^
CENTRAL_NERVOUS_SYSTEM_DEVELOPMENT	980	19	4.77 × 10^−7^
MODULE_137	545	14	6.47 × 10^−7^
MODULE_100	543	14	6.19 × 10^−7^
MODULE_66	551	14	7.36 × 10^−7^
HALLMARK_ANGIOGENESIS	36	5	9.28 × 10^−7^

**Table 2:** The Top 20 significantly enriched MSigDB pathways associated with the specific lists of genes down-regulated in GASC-LS. The table reports the overall number (#) of genes involved in the annotated pathways, the number (#)of genes provided as input, and the associated *p*-value.

GASC-LS DOWN			
Gene Set Name	#Genes in Gene Set	#Genes in the Overlap	*p*-Value
CELL_CELL_SIGNALING	1672	21	5.52 × 10^−8^
MODULE_88	834	14	3.84 × 10^−7^
MODULE_55	831	14	3.67 × 10^−7^
ANTI_TREM1_VS_ANTI_TREM1_AND_LPS_MONOCYTE_DN	195	8	2.00 × 10^−7^
UNTREATED_VS_IL2_TREATED_STAT5_AB_KNOCKIN_TCELL_ 2H_UP	200	8	2.43 × 10^−7^
MODULE_64	517	11	7.49 × 10^−7^
DEFENSE_RESPONSE	1790	20	7.52 × 10^−7^
MODULE_24	453	10	1.76 × 10^−6^
REGULATION_OF_TRANSPORT	1730	19	1.89 × 10^−6^
LOW_LPS_VS_VEHICLE_TREATED_MONOCYTE_UP	196	7	2.99 × 10^−6^
ANTI_TREM1_AND_LPS_VS_VEHICLE_TREATED_MONOCYTES_UP	195	7	2.89 × 10^−6^
NEUTROPHIL_VS_DC_UP	199	7	3.30 × 10^−6^
REACTOME_DNA_DAMAGE_TELOMERE_STRESS_INDUCED_ SENESCENCE	80	5	5.49 × 10^−6^
MODULE_89	14	3	1.06 × 10^−5^
REGULATION_OF_IMMUNE_SYSTEM_PROCESS	1593	17	1.01 × 10^−5^
REACTOME_SIGNALING_BY_INTERLEUKINS	463	9	1.62 × 10^−5^
MODULE_203	16	3	1.62 × 10^−5^
SYNAPTIC_SIGNALING	712	11	1.56 × 10^−5^
REGULATION_OF_ION_TRANSPORT	1314	15	1.61 × 10^−5^
MODULE_90	17	3	1.96 × 10^−5^

**Table 3:** Comparison of genes differentially expressed in GASC-LS versus GASC-SS. The table shows genes with a significant down-regulation in GASC-LS compared to GASC-SS. Values indicate Fold Regulation calculated using the formula (2^(−ΔΔCT)^), i.e., the normalized gene expression (2^(−ΔCT)^) in the test sample (GASC-LS) divided by the normalized gene expression (2^(−ΔCT)^) in the control sample (GASC-SS). *p*-values are calculated based on a Student’s t-test of the replicate 2^(−ΔCT)^ values for each gene in the control and test group.

Gene Symbol	Gene Name	Fold Change	*p*-Value
VGF	VGF nerve growth factor inducible	−5.97	2.30 × 10^−2^
ALPL	Alkaline Phosphatase, liver/bone/Kidney	−5.7	1.50 × 10^−2^
GPR68	G protein coupled receptor 68	−2.61	1.80 × 10^−2^
NETO1	Neuropilin and tolloid-like 1	−3.52	1.30 × 10^−2^

## Data Availability

The RNAseq data are available as GEO accession GSE199407 Go to https://www.ncbi.nlm.nih.gov/geo/query/acc.cgi?acc=GSE199407. Enter token evqzukemxzgfhap into the box.

## Supplementary Materials

The following supporting information can be downloaded at: https://www.mdpi.com/article/10.3390/cancers14143413/s1, Table S1: Clinical characteristics of patients from which GASC-LS and GASC-SS were isolated; Table S2: Gene expression profile of GASC-LS and GASC-SS; Figure S1: Clinical relevance of the 4 genes down-regulated in GASC-LS.

## References

[1] Stupp R., Mason W.P., van den Bent M.J., Weller M., Fisher B., Taphoorn M.J.B., Belanger K., Brandes A.A., Marosi C., Bogdahn U. (2005). Radiotherapy plus Concomitant and Adjuvant Temozolomide for Glioblastoma. N. Engl. J. Med..

[2] Ostrom Q.T., Gittleman H., Liao P., Rouse C., Chen Y., Dowling J., Wolinsky Y., Kruchko C., Barnholtz-Sloan J. (2014). CBTRUS Statistical Report: Primary Brain and Central Nervous System Tumors Diagnosed in the United States in 2007–2011. Neuro Oncol..

[3] Lara-Velazquez M., Al-Kharboosh R., Jeanneret S., Vazquez-Ramos C., Mahato D., Tavanaiepour D., Rahmathulla G., Quinones-Hinojosa A. (2017). Advances in Brain Tumor Surgery for Glioblastoma in Adults. Brain Sci..

[4] Quail D.F., Joyce J.A. (2013). Microenvironmental Regulation of Tumor Progression and Metastasis. Nat. Med..

[5] Jhaveri N., Chen T.C., Hofman F.M. (2016). Tumor Vasculature and Glioma Stem Cells: Contributions to Glioma Progression. Cancer Lett..

[6] Lathia J.D., Heddleston J.M., Venere M., Rich J.N. (2011). Deadly Teamwork: Neural Cancer Stem Cells and the Tumor Microenvironment. Cell Stem Cell.

[7] Current Treatments for Brain Tumors. https://www.bing.com/search?q=Current+Treatments+for+Brain+Tumors.+Available+online%3A+https%3A%2F%2Fbraintumor.org.

[8] Drugs Approved for Brain Tumors—National Cancer Institute. https://www.cancer.gov/about-cancer/treatment/drugs/brain.

[9] Bota D.A., Desjardins A., Quinn J.A., Affronti M.L., Friedman H.S. (2007). Interstitial Chemotherapy with Biodegradable BCNU (Gliadel) Wafers in the Treatment of Malignant Gliomas. Ther. Clin. Risk Manag..

[10] Shibahara I., Hanihara M., Watanabe T., Dan M., Sato S., Kuroda H., Inamura A., Inukai M., Hara A., Yasui Y. (2018). Tumor Microenvironment after Biodegradable BCNU Wafer Implantation: Special Consideration of Immune System. J. Neurooncol..

[11] Han S., Zhang C., Li Q., Dong J., Liu Y., Huang Y., Jiang T., Wu A. (2014). Tumour-Infiltrating CD4+ and CD8+ Lymphocytes as Predictors of Clinical Outcome in Glioma. Br. J. Cancer.

[12] Brem H., Tamargo R.J., Olivi A., Pinn M., Weingart J.D., Wharam M., Epstein J.I. (1994). Biodegradable Polymers for Controlled Delivery of Chemotherapy with and without Radiation Therapy in the Monkey Brain. J. Neurosurg..

[13] Xing W., Shao C., Qi Z., Yang C., Wang Z. (2015). The Role of Gliadel Wafers in the Treatment of Newly Diagnosed GBM: A Meta-Analysis. Drug Des. Dev. Ther..

[14] Ashby L.S., Smith K.A., Stea B. (2016). Gliadel Wafer Implantation Combined with Standard Radiotherapy and Concurrent Followed by Adjuvant Temozolomide for Treatment of Newly Diagnosed High-Grade Glioma: A Systematic Literature Review. World J. Surg. Oncol..

[15] Bregy A., Shah A.H., Diaz M.V., Pierce H.E., Ames P.L., Diaz D., Komotar R.J. (2013). The Role of Gliadel Wafers in the Treatment of High-Grade Gliomas. Expert Rev. Anticancer Ther..

[16] Westphal M., Hilt D.C., Bortey E., Delavault P., Olivares R., Warnke P.C., Whittle I.R., Jääskeläinen J., Ram Z. (2003). A Phase 3 Trial of Local Chemotherapy with Biodegradable Carmustine (BCNU) Wafers (Gliadel Wafers) in Patients with Primary Malignant Glioma. Neuro Oncol..

[17] Attenello F.J., Mukherjee D., Datoo G., McGirt M.J., Bohan E., Weingart J.D., Olivi A., Quinones-Hinojosa A., Brem H. (2008). Use of Gliadel (BCNU) Wafer in the Surgical Treatment of Malignant Glioma: A 10-Year Institutional Experience. Ann. Surg. Oncol..

[18] McGirt M.J., Than K.D., Weingart J.D., Chaichana K.L., Attenello F.J., Olivi A., Laterra J., Kleinberg L.R., Grossman S.A., Brem H. (2009). Gliadel (BCNU) Wafer plus Concomitant Temozolomide Therapy after Primary Resection of Glioblastoma Multiforme. J. Neurosurg..

[19] Ius T., Cesselli D., Isola M., Toniato G., Pauletto G., Sciacca G., Fabbro S., Pegolo E., Rizzato S., Beltrami A.P. (2018). Combining Clinical and Molecular Data to Predict the Benefits of Carmustine Wafers in Newly Diagnosed High-Grade Gliomas. Curr. Treat. Options Neurol..

[20] National Research Council (US) Committee on a Framework for Developing a New Taxonomy of Disease (2011). Toward Precision Medicine: Building a Knowledge Network for Biomedical Research and a New Taxonomy of Disease.

[21] Eckel-Passow J.E., Lachance D.H., Molinaro A.M., Walsh K.M., Decker P.A., Sicotte H., Pekmezci M., Rice T., Kosel M.L., Smirnov I.V. (2015). Glioma Groups Based on 1p/19q, IDH, and TERT Promoter Mutations in Tumors. N. Engl. J. Med..

[22] Brat D.J., Verhaak R.G.W., Aldape K.D., Yung W.K.A., Salama S.R., Cooper L.A.D., Rheinbay E., Miller C.R., Vitucci M., Cancer Genome Atlas Research Network (2015). Comprehensive, Integrative Genomic Analysis of Diffuse Lower-Grade Gliomas. N. Engl. J. Med..

[23] Ceccarelli M., Barthel F.P., Malta T.M., Sabedot T.S., Salama S.R., Murray B.A., Morozova O., Newton Y., Radenbaugh A., Pagnotta S.M. (2016). Molecular Profiling Reveals Biologically Discrete Subsets and Pathways of Progression in Diffuse Glioma. Cell.

[24] Sankar P.L., Parker L.S. (2017). The Precision Medicine Initiative’s All of Us Research Program: An Agenda for Research on Its Ethical, Legal, and Social Issues. Genet Med.

[25] Hanahan D., Weinberg R.A. (2011). Hallmarks of Cancer: The next Generation. Cell.

[26] Beltrami A.P., Cesselli D., Bergamin N., Marcon P., Rigo S., Puppato E., D’Aurizio F., Verardo R., Piazza S., Pignatelli A. (2007). Multipotent Cells Can Be Generated in Vitro from Several Adult Human Organs (Heart, Liver, and Bone Marrow). Blood.

[27] Bourkoula E., Mangoni D., Ius T., Pucer A., Isola M., Musiello D., Marzinotto S., Toffoletto B., Sorrentino M., Palma A. (2014). Glioma-Associated Stem Cells: A Novel Class of Tumor-Supporting Cells Able to Predict Prognosis of Human Low-Grade Gliomas. Stem Cells.

[28] Manini I., Ruaro M.E., Sgarra R., Bartolini A., Caponnetto F., Ius T., Skrap M., Di Loreto C., Beltrami A.P., Manfioletti G. (2019). Semaphorin-7A on Exosomes: A Promigratory Signal in the Glioma Microenvironment. Cancers.

[29] Ius T., Ciani Y., Ruaro M.E., Isola M., Sorrentino M., Bulfoni M., Candotti V., Correcig C., Bourkoula E., Manini I. (2018). An NF-ΚB Signature Predicts Low-Grade Glioma Prognosis: A Precision Medicine Approach Based on Patient-Derived Stem Cells. Neuro Oncol..

[30] Manini I., Caponnetto F., Dalla E., Ius T., Della Pepa G.M., Pegolo E., Bartolini A., La Rocca G., Menna G., Di Loreto C. (2020). Heterogeneity Matters: Different Regions of Glioblastoma Are Characterized by Distinctive Tumor-Supporting Pathways. Cancers.

[31] Caponnetto F., Dalla E., Mangoni D., Piazza S., Radovic S., Ius T., Skrap M., Di Loreto C., Beltrami A.P., Manini I. (2020). The MiRNA Content of Exosomes Released from the Glioma Microenvironment Can Affect Malignant Progression. Biomedicines.

[32] Babraham Bioinformatics—FastQC a Quality Control Tool for High Throughput Sequence Data. https://www.bioinformatics.babraham.ac.uk/projects/fastqc/.

[33] Ewels P., Magnusson M., Lundin S., Käller M. (2016). MultiQC: Summarize Analysis Results for Multiple Tools and Samples in a Single Report. Bioinformatics.

[34] Bolger A.M., Lohse M., Usadel B. (2014). Trimmomatic: A Flexible Trimmer for Illumina Sequence Data. Bioinformatics.

[35] Dobin A., Davis C.A., Schlesinger F., Drenkow J., Zaleski C., Jha S., Batut P., Chaisson M., Gingeras T.R. (2013). STAR: Ultrafast Universal RNA-Seq Aligner. Bioinformatics.

[36] Pertea M., Pertea G.M., Antonescu C.M., Chang T.-C., Mendell J.T., Salzberg S.L. (2015). StringTie Enables Improved Reconstruction of a Transcriptome from RNA-Seq Reads. Nat. Biotechnol..

[37] Love M.I., Huber W., Anders S. (2014). Moderated Estimation of Fold Change and Dispersion for RNA-Seq Data with DESeq2. Genome Biol..

[38] Durinck S., Spellman P.T., Birney E., Huber W. (2009). Mapping Identifiers for the Integration of Genomic Datasets with the R/Bioconductor Package BiomaRt. Nat. Protoc..

[39] Zhang Y.-D., Dai R.-Y., Chen Z., Zhang Y.-H., He X.-Z., Zhou J. (2014). Efficacy and Safety of Carmustine Wafers in the Treatment of Glioblastoma Multiforme: A Systematic Review. Turk. Neurosurg..

[40] Gutenberg A., Lumenta C.B., Braunsdorf W.E.K., Sabel M., Mehdorn H.M., Westphal M., Giese A. (2013). The Combination of Carmustine Wafers and Temozolomide for the Treatment of Malignant Gliomas. A Comprehensive Review of the Rationale and Clinical Experience. J. Neurooncol..

[41] Catacuzzeno L., Franciolini F. (2018). Role of KCa3.1 Channels in Modulating Ca^2+^ Oscillations during Glioblastoma Cell Migration and Invasion. Int. J. Mol. Sci..

[42] Cuddapah V.A., Turner K.L., Seifert S., Sontheimer H. (2013). Bradykinin-Induced Chemotaxis of Human Gliomas Requires the Activation of KCa3.1 and ClC-3. J. Neurosci..

[43] Venkatesh H.S., Morishita W., Geraghty A.C., Silverbush D., Gillespie S.M., Arzt M., Tam L.T., Espenel C., Ponnuswami A., Ni L. (2019). Electrical and Synaptic Integration of Glioma into Neural Circuits. Nature.

[44] Monje M., Borniger J.C., D’Silva N.J., Deneen B., Dirks P.B., Fattahi F., Frenette P.S., Garzia L., Gutmann D.H., Hanahan D. (2020). Roadmap for the Emerging Field of Cancer Neuroscience. Cell.

[45] Panyi G., Beeton C., Felipe A. (2014). Ion Channels and Anti-Cancer Immunity. Philos. Trans. R. Soc. Lond B Biol. Sci..

[46] Kale V.P., Amin S.G., Pandey M.K. (2015). Targeting Ion Channels for Cancer Therapy by Repurposing the Approved Drugs. Biochim Biophys. Acta.

[47] Bagal S.K., Brown A.D., Cox P.J., Omoto K., Owen R.M., Pryde D.C., Sidders B., Skerratt S.E., Stevens E.B., Storer R.I. (2013). Ion Channels as Therapeutic Targets: A Drug Discovery Perspective. J. Med. Chem..

[48] Arvanitis C.D., Ferraro G.B., Jain R.K. (2020). The Blood-Brain Barrier and Blood-Tumour Barrier in Brain Tumours and Metastases. Nat. Rev. Cancer.

[49] Alphandéry E. (2020). Nano-Therapies for Glioblastoma Treatment. Cancers.

[50] Takahashi K., Tanabe K., Ohnuki M., Narita M., Ichisaka T., Tomoda K., Yamanaka S. (2007). Induction of Pluripotent Stem Cells from Adult Human Fibroblasts by Defined Factors. Cell.

[51] Rico L.G., Juncà J., Ward M.D., Bradford J., Petriz J. (2016). Is Alkaline Phosphatase the Smoking Gun for Highly Refractory Primitive Leukemic Cells?. Oncotarget.

[52] Vassilatis D.K., Hohmann J.G., Zeng H., Li F., Ranchalis J.E., Mortrud M.T., Brown A., Rodriguez S.S., Weller J.R., Wright A.C. (2003). The G Protein-Coupled Receptor Repertoires of Human and Mouse. Proc. Natl. Acad. Sci. USA.

[53] Lynch J.R., Wang J.Y. (2016). G Protein-Coupled Receptor Signaling in Stem Cells and Cancer. Int. J. Mol. Sci..

[54] O’Hayre M., Degese M.S., Gutkind J.S. (2014). Novel Insights into G Protein and G Protein-Coupled Receptor Signaling in Cancer. Curr. Opin. Cell Biol..

[55] Vaupel P., Kallinowski F., Okunieff P. (1989). Blood Flow, Oxygen and Nutrient Supply, and Metabolic Microenvironment of Human Tumors: A Review. Cancer Res..

[56] Gatenby R.A., Gillies R.J. (2004). Why Do Cancers Have High Aerobic Glycolysis?. Nat. Rev. Cancer.

[57] Cairns R., Papandreou I., Denko N. (2006). Overcoming Physiologic Barriers to Cancer Treatment by Molecularly Targeting the Tumor Microenvironment. Mol. Cancer Res..

[58] Damaghi M., Wojtkowiak J.W., Gillies R.J. (2013). PH Sensing and Regulation in Cancer. Front. Physiol..

[59] Justus C.R., Dong L., Yang L.V. (2013). Acidic Tumor Microenvironment and PH-Sensing G Protein-Coupled Receptors. Front. Physiol..

[60] Insel P.A., Sriram K., Wiley S.Z., Wilderman A., Katakia T., McCann T., Yokouchi H., Zhang L., Corriden R., Liu D. (2018). GPCRomics: GPCR Expression in Cancer Cells and Tumors Identifies New, Potential Biomarkers and Therapeutic Targets. Front. Pharmacol..

[61] Wiley S.Z., Sriram K., Liang W., Chang S.E., French R., McCann T., Sicklick J., Nishihara H., Lowy A.M., Insel P.A. (2018). GPR68, a Proton-Sensing GPCR, Mediates Interaction of Cancer-Associated Fibroblasts and Cancer Cells. FASEB J..

[62] Zhang W., St-Gelais F., Grabner C.P., Trinidad J.C., Sumioka A., Morimoto-Tomita M., Kim K.S., Straub C., Burlingame A.L., Howe J.R. (2009). A Transmembrane Accessory Subunit That Modulates Kainate-Type Glutamate Receptors. Neuron.

[63] Copits B.A., Swanson G.T. (2012). Dancing Partners at the Synapse: Auxiliary Subunits That Shape Kainate Receptor Function. Nat. Rev. Neurosci..

[64] Copits B.A., Robbins J.S., Frausto S., Swanson G.T. (2011). Synaptic Targeting and Functional Modulation of GluK1 Kainate Receptors by the Auxiliary Neuropilin and Tolloid-like (NETO) Proteins. J. Neurosci..

[65] Straub C., Hunt D.L., Yamasaki M., Kim K.S., Watanabe M., Castillo P.E., Tomita S. (2011). Distinct Functions of Kainate Receptors in the Brain Are Determined by the Auxiliary Subunit Neto1. Nat. Neurosci..

[66] Bowie D. (2008). Ionotropic Glutamate Receptors & CNS Disorders. CNS Neurol. Disord. Drug Targets.

[67] Vincent P., Mulle C. (2009). Kainate Receptors in Epilepsy and Excitotoxicity. Neuroscience.

[68] Xu Y., Wang W., Chen J., Mao H., Liu Y., Gu S., Liu Q., Xi Q., Shi W. (2020). High Neuropilin and Tolloid-like 1 Expression Associated with Metastasis and Poor Survival in Epithelial Ovarian Cancer via Regulation of Actin Cytoskeleton. J. Cell. Mol. Med..

[69] Levi A., Eldridge J.D., Paterson B.M. (1985). Molecular Cloning of a Gene Sequence Regulated by Nerve Growth Factor. Science.

[70] Levi A., Ferri G.-L., Watson E., Possenti R., Salton S.R.J. (2004). Processing, Distribution, and Function of VGF, a Neuronal and Endocrine Peptide Precursor. Cell. Mol. Neurobiol..

[71] Watson E., Fargali S., Okamoto H., Sadahiro M., Gordon R.E., Chakraborty T., Sleeman M.W., Salton S.R. (2009). Analysis of Knockout Mice Suggests a Role for VGF in the Control of Fat Storage and Energy Expenditure. BMC Physiol..

[72] Hunsberger J.G., Newton S.S., Bennett A.H., Duman C.H., Russell D.S., Salton S.R., Duman R.S. (2007). Antidepressant Actions of the Exercise-Regulated Gene VGF. Nat. Med..

[73] Hwang W., Chiu Y.-F., Kuo M.-H., Lee K.-L., Lee A.-C., Yu C.-C., Chang J.-L., Huang W.-C., Hsiao S.-H., Lin S.-E. (2017). Expression of Neuroendocrine Factor VGF in Lung Cancer Cells Confers Resistance to EGFR Kinase Inhibitors and Triggers Epithelial-to-Mesenchymal Transition. Cancer Res..

[74] Mani S.A., Guo W., Liao M.-J., Eaton E.N., Ayyanan A., Zhou A.Y., Brooks M., Reinhard F., Zhang C.C., Shipitsin M. (2008). The Epithelial-Mesenchymal Transition Generates Cells with Properties of Stem Cells. Cell.

[75] Wang X., Prager B.C., Wu Q., Kim L.J.Y., Gimple R.C., Shi Y., Yang K., Morton A.R., Zhou W., Zhu Z. (2018). Reciprocal Signaling between Glioblastoma Stem Cells and Differentiated Tumor Cells Promotes Malignant Progression. Cell Stem Cell.

