# Supplementary material for: Identification of a Prognostic Microenvironment-Related Gene Signature in Glioblastoma Patients Treated with Carmustine Wafers
Source: Cancers (Basel). 2022 Jul 14;14(14):3413. doi: 10.3390/cancers14143413 (PMC9320240; doi:10.3390/cancers14143413)
Supplement: Supplementary file 1 [file cancers-14-03413-s001.zip › Supplementary/Caption Supplementary.docx]

**Supplementary Table S1.**

Clinical characteristics of patients from which GASC-LS and GASC-SS analysed in the study were isolated. In the table were reported the IDH1 status, the age, the extent of resection at surgery (%), the Overall Survival (OS), the Progression Free Survival (PFS) and the therapeutic protocol.

**Supplementary Table S2.**

Gene expression profile of GASC-LS and GASC-SS. The sheet named “Whole” represents the entire genomic signature of the two cell populations (expressed and not expressed transcripts, significantly or not significantly differentially expressed transcripts). In the sheet named “allgenes_abs(LFC)>1_Padj0.05” were reported the significantly differentially up- and down-regulated transcripts in GASC-LS compared with GASC-SS.

**Supplementary Figure 1.**

Clinical relevance of the 4 genes down-regulated in GASC-LS. The prognostic value of ALPL, GPR68, VGF and NETO1 was assessed independently in the TCGA-GBM RNA-seq dataset evaluating the Progression Free Survival (PFS) and was represented by Kaplan-Meier plots. Afterwards, the analysis was repeated considering the four-genes signature (median expression). Patients were stratified based on the optimal cut-point.
