# Supplementary material for: Identification of a Prognostic Microenvironment-Related Gene Signature in Glioblastoma Patients Treated with Carmustine Wafers
Source: Cancers (Basel). 2022 Jul 14;14(14):3413. doi: 10.3390/cancers14143413 (PMC9320240; doi:10.3390/cancers14143413)
Supplement: Supplementary file 1 [file cancers-14-03413-s001.zip › Supplementary/Supplementary table S1.docx]

| PATIENTS | DIAGNOSIS | AGE | EOR (%) | OS (months) | PFS (months) | THERAPY |
| --- | --- | --- | --- | --- | --- | --- |
| GASC-LS_1 | GBM-IDH1 wt | 62 | 100 | 14 | 6 | CW+Stupp |
| GASC-LS_2 | GBM-IDH1 wt | 65 | 100 | 12 | 8 | CW+Stupp |
| GASC-LS_3 | GBM-IDH1 wt | 65 | 85 | 13 | 4 | CW+Stupp |
| GASC-LS_4 | GBM-IDH1 wt | 55 | 100 | 12 | 8 | CW+Stupp |
| GASC-LS_5 | GBM-IDH1 wt | 30 | 100 | 14 | 4 | CW+Stupp |
| GASC-LS_6 | GBM-IDH1 wt | 55 | 100 | 40 | 32 | CW+Stupp |
| GASC-SS_1 | GBM-IDH1 wt | 37 | 100 | 40 | 33 | CW+Stupp |
| GASC-SS_2 | GBM-IDH1 wt | 58 | 100 | 36 | 36 | CW+Stupp |
| GASC-SS_3 | GBM-IDH1 wt | 69 | 100 | 43 | 33 | CW+Stupp |
| GASC-SS_4 | GBM-IDH1 wt | 65 | 100 | 30 | 30 | CW+Stupp |
| GASC-SS_5 | GBM-IDH1 wt | 54 | 100 | 41 | 41 | CW+Stupp |
|  |  |  |  |  |  |  |
